# Serum Folate Status Is Primarily Associated With Neurodevelopment in Children With Autism Spectrum Disorders Aged Three and Under—A Multi-Center Study in China

**DOI:** 10.3389/fnut.2021.661223

**Published:** 2021-05-13

**Authors:** Qiu Li, Ting Yang, Li Chen, Ying Dai, Li-Jie Wu, Fei-Yong Jia, Yan Hao, Ling Li, Jie Zhang, Xiao-Yan Ke, Ming-Ji Yi, Qi Hong, Jin-Jin Chen, Shuan-Feng Fang, Yi-Chao Wang, Qi Wang, Chun-Hua Jin, Zhi-Fang Dong, Jie Chen, Ting-Yu Li

**Affiliations:** ^1^Chongqing Key Laboratory of Childhood Nutrition and Health, Children's Hospital of Chongqing Medical University, Ministry of Education Key Laboratory of Child Development and Disorders, National Clinical Research Center for Child Health and Disorders, Chongqing, China; ^2^Department of Children's and Adolescent Health, Public Health College of Harbin Medical University, Harbin, China; ^3^Department of Developmental and Behavioral Pediatrics, The First Hospital of Jilin University, Changchun, China; ^4^Department of Pediatrics, Tongji Hospital, Tongji Medical College, Huazhong University of Science and Technology, Wuhan, China; ^5^Department of Children Rehabilitation, Hainan Women and Children's Medical Center, Haikou, China; ^6^Children Health Care Center, Xi'an Children's Hospital, Xi'an, China; ^7^Child Mental Health Research Center of Nanjing Brain Hospital, Nanjing, China; ^8^Department of Child Health Care, The Affiliated Hospital of Qingdao University, Qingdao, China; ^9^Maternal and Child Health Hospital of Baoan, Shenzhen, China; ^10^Department of Child Healthcare, Shanghai Children's Hospital, Shanghai Jiao Tong University, Shanghai, China; ^11^Children's Hospital Affiliated to Zhengzhou University, Zhengzhou, China; ^12^National Health Commission Key Laboratory of Birth Defect for Research and Prevention, Hunan Provincial Maternal and Child Health Care Hospital, Changsha, China; ^13^Deyang Maternity & Child Healthcare Hospital, Deyang, China; ^14^Department of Children Health Care, Capital Institute of Pediatrics, Beijing, China

**Keywords:** autism, folate, early life, neurodevelopment, children

## Abstract

**Background:** Autism spectrum disorder (ASD) is a complex neurodevelopmental disorder. Folate has been demonstrated to be associated with ASD. However, current studies on the correlation between folate and symptoms of children with ASD have inconsistent conclusions, use mainly small samples, and lack age-stratified analysis. This study aimed to explore the association between serum folate and symptoms of autistic children at different age groups from a multi-center perspective.

**Methods:** We enrolled 1,300 children with ASD and 1,246 typically developing (TD) children under 7 years old from 13 cities in China. The Autism Behavior Checklist (ABC), Social Responsiveness Scale (SRS), and Childhood autism rating scale (CARS) were used to evaluate the symptoms of children with ASD. China neuropsychological and Behavior Scale-Revision 2016 (CNBS-R2016) scale was used to evaluate the neurodevelopment of children with ASD. Serum folate was measured by chemiluminescence assay in the two groups.

**Results:** The serum folate levels of children with ASD were lower than that of TD children. In terms of core symptoms of ASD, we found that the serum folate levels were not associated with ABC, SRS, and CARS scores in ASD children of all ages but negatively associated with communication warning behavior scores of CNBS-R2016 in ASD children aged three and under. Concerning development quotients, it was at the age of three and under that serum folate levels were positively associated with gross motor, fine motor, language, and general quotient of ASD children. These ASD children aged three and under were further divided into two groups according to the median of serum folate (14.33 ng/mL); we found that compared to ASD children with folate ≤ 14.33 ng/mL, those with folate >14.33 ng/mL had lower communication warning behavior score and higher gross motor, fine motor, adaptive behavior, language, person-social, and general development quotients.

**Conclusion:** We found that serum folate status was primarily associated with the neurodevelopment of children with ASD aged three and under. Furthermore, relatively higher serum folate levels may be more beneficial for children with ASD. Our results suggest that folate level should be paid more attention in ASD children, especially in early life, to better promote the intervention of ASD children.

## Introduction

Autism spectrum disorder (ASD) is a developmental disability characterized by persistent impairments in social interaction and the presence of restricted, repetitive patterns of behaviors, interests, or activities (1). Recently, the prevalence of autism has increased steeply. According to the latest estimation done by the American Center for Disease Control and Prevention, on average, 1 out of 54 children aged 8 has been identified with ASD (2). The data from China in 2019 also showed that the prevalence rate of children with ASD aged 6–12 can be as high as roughly 0.7% (3). This increasing trend has brought an enormous burden on families and society (1).

The etiology of ASD is complex. Genetic (4, 5) and environmental factors (6–9) may play a key role in the pathogenesis of ASD. Nutrients are important environmental factors. Many studies have found that micronutrient levels of children with ASD differ from those of normal children (10). For instance, folate (vitamin B9) plays critical roles in the nervous system, including normal brain development and maintenance function (11). Multiple recent studies demonstrated that folic acid supplementation in pregnancy, as well as the blood and urine folate levels, is correlated with the incidence of ASD (12). Several studies have reported that blood and urine folate levels of children with ASD were lower than that in typically developing (TD) children (13, 14). Our previous surveys on serum folate between autistic children and control children showed the same result.

The correlation between folate level and ASD symptoms has also been studied by some researchers. Alun et al. reported that serum folate level was reduced in children with ASD, and serum folate level was negatively associated with the severity of ASD core symptoms when assessed by the Childhood autism rating scale (CARS) (13). However, another study found that the serum folate levels were not associated with the CARS scores of ASD children (15). Our previous single-center study showed that serum folate was mainly associated with the development quotient rather than scores on the Autism Behavior Checklist (ABC) and CARS of ASD children (16). These inconsistent conclusions may be due to the different ethnicities, different symptom assessment scales, single-center research, small sample sizes, and so on. Therefore, large-sample and multi-center studies are needed to further explore whether folate is more closely related to the core symptoms or neurodevelopmental levels of children with ASD. This is especially important as the critical period of a child's brain is between pregnancy and 3 years of age (17–19), and brain development is most easily affected by external environmental factors during this period (20). Hence, researchers suggest that the earlier ASD children receive behavioral intervention, the more likely they are to improve symptoms (21, 22). For example, the study of Ramaekers et al. showed that the degree of improvement of ASD symptoms by folic acid treatment decreased with age (23). Therefore, the association of folate with core symptoms and neurodevelopment levels in children with ASD may vary in different age groups. However, there is still no study focusing on the relationship between folate and core symptoms and neurodevelopment levels of ASD children after age stratification.

Given this background, in this paper, our overarching goal was to determine the associations between serum folate status and core symptoms and neurodevelopmental levels of ASD children at different age groups from a multi-center perspective. Within this goal, we pursued two aims. First, we investigated the serum folate status between ASD children and TD children. Second, we sought to understand the serum folate effect on ASD symptoms and developmental levels at different age stages.

## Methods

### Study Population

**Figure 1:**
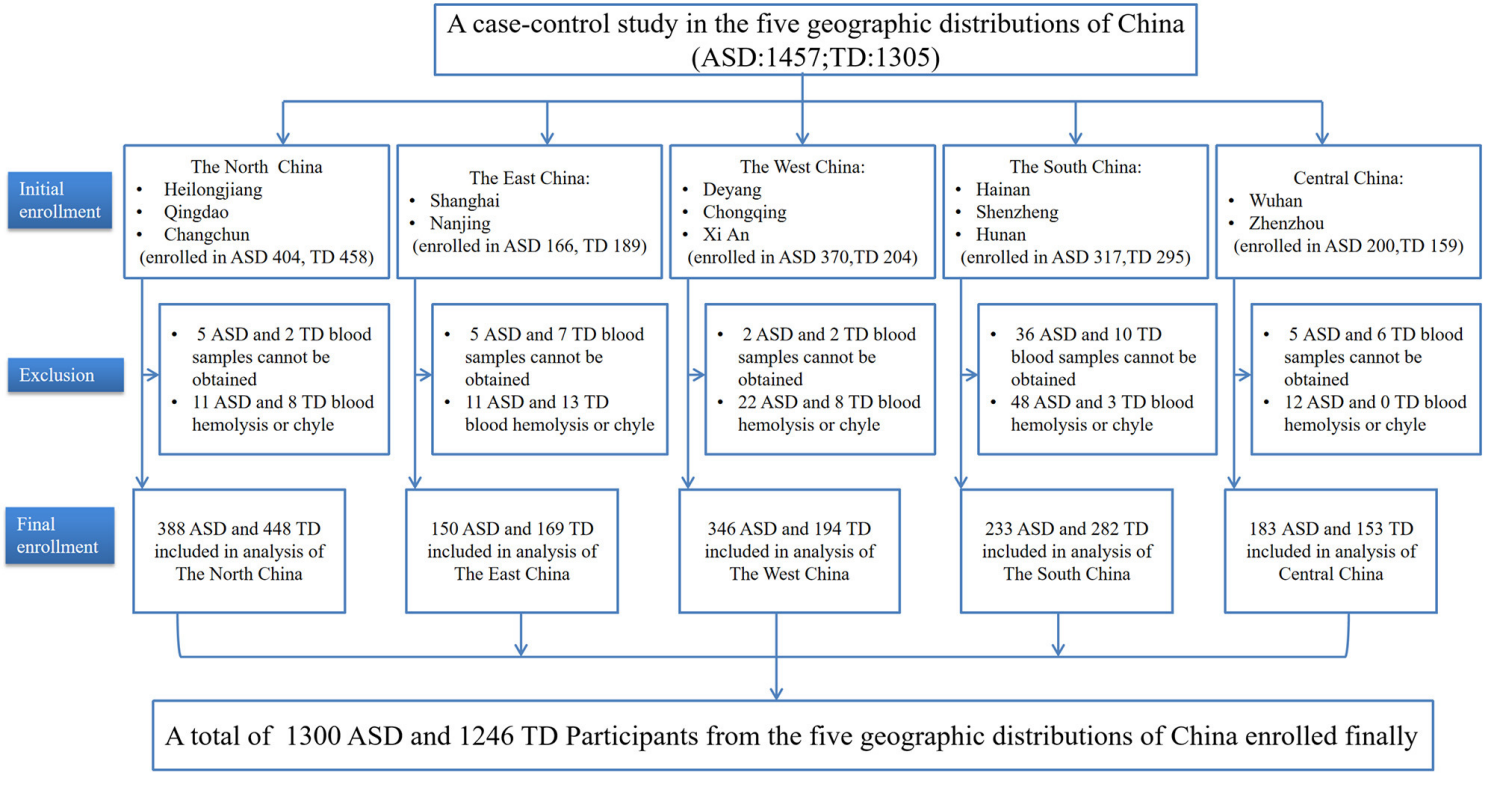
Flowchart of the study participants: a case-control design, and multi-center study in China. ASD, Autism spectrum disorder; TD, typically developing.

In this multi-center study, participants were recruited from May 2018 to December 2019 across 13 cities in five geographic areas of China (participating centers and the enrollment of the subjects are shown in Figure 1). A total of 2,546 children under 7 years old, including 1,457 ASD children and 1,305 typically developing (TD) children, participated in the study. Among these participants, 1,404 ASD children and 1,278 TD children's blood samples were obtained. Because hemolysis or chylous occurred in blood samples of 104 ASD children and 32 TD children, these children were excluded from the analysis. As a result, the final sample size consisted of 2,546 children, including 1,300 ASD children and 1,246 TD children. ASD children were recruited from a variety of sources including hospital rehabilitation or training centers and special needs schools. Children with a DSM-5 clinical diagnosis of autism spectrum disorder by an experienced developmental pediatrician and a psychologist of the children's hospital at each center were included in the study. The exclusion criteria were as follows: a history of other developmental disorders or psychiatric diseases, such as Rett Syndrome, cerebral palsy, chronic seizures, and other congenital diseases; a history of infection during the previous 3 months; and the use of any folate supplementation in the previous 3 months. The control group was recruited from local preschools and online registration without a history of motor or language impairments, or social developmental disorders, according to the reports of their parents and teachers. None of the children in the control group had an acute or chronic infectious disease in the past 3 months, and none of these children received any folate supplementation during the previous 3 months.

All participants volunteered for this study. Informed consent was provided by the primary caregiver. The study protocol was approved by the ethics committee of the Children's Hospital of Chongqing Medical University, Approval Number: (2018) IRB (STUDY) NO. 121 and registered in the Chinese Clinical Trial Registry (ChiCTR) (Registration number: ChiCTR2000031194).

### Clinical Measures of Children With ASD

The caregivers of the ASD and control children completed the information questionnaires including questions concerning demographic data (e.g., name, age, and gender), medical history, and the preference of foods. Symptoms of ASD were assessed with the Autism Behavior Checklist (ABC), Social Responsiveness Scale (SRS), and Childhood Autism Rating Scale (CARS). The ABC is a behavior rating scale used to measure behavior problems across five subscales: sensory, relating, stereotypic behavior, language, and social self-help,. Scores for children without autism should be <53 (24). The SRS is an assessment scale of social impairment within autism across five subscales, including social awareness, social communication, social motivation, and autistic mannerisms. Typically, the SRS score for normal children should be <65 (25). The CARS is an assessment scale of the severity of autistic symptomatology. It consists of 15 items that are rated on a four-point scale. Children with scores between 30 and 36 are considered as having mild-moderate autism and scores between 37 and 60 are considered as severe autism (26). Neurodevelopmental levels of children with ASD were evaluated with Children Neuropsychological and Behavior Scale-Revision 2016 (CNBS-R2016) in China. The CNBS-R2016 is a diagnostic assessment tool developed by the Capital Institute of Pediatrics in China. The CNBS-R2016 and the Griffiths Mental Development Scales (GDS-C) tests have good consistency in the developmental assessment of children with ASD (27). It includes six subscales: gross motor, fine motor, adaptive behavior, personal-social, language, and communication warning behavior. The communication warning behavior is an independent subscale and an assessment of autism symptoms. A general or subscale quotient <70 points [ <2 standard deviations (SDs)] indicates a developmental delay (DD). For the subscale of communication warning behavior, a quotient <7 points indicates typical development; a quotient between 7 and 12 points indicates a need for follow-up. A quotient between 12 and 30 points indicates a risk of communication and interaction disorder; a quotient >30 points indicates a high suspicion of ASD. In our study, the number of valid ABC, SRS, and CARS were 1215, 1108, and 1105, respectively. The valid number for CNBS-R2016 was 934.

### Laboratory Measurements

Five milliliters (collected by a dry and additive-free tube) of venous blood were drawn from the participant's median cubital vein by venipuncture. After centrifugation at room temperature at 3,000 rpm for 10 min, the serum was transferred to a microcentrifuge tube and stored at −80°C before the measurements of folate. Automatic chemiluminescence immunoassay (mindray, CL-1000i, China) was used to quantitatively determine the serum level of folate.

### Statistical Analysis

The Kolmogorov–Smirnov goodness-of-fit test was used to test the distribution of each dataset for normality before analysis. Continuous variables are presented as the means (with SEM) or medians (with interquartile ranges, IQR). Categorical variables were described as *N* (%). Differences among groups were assessed by using the Chi-Square test, Mann-Whitney test, and Kruskal-Wallis with the Bonferroni *post hoc* test. Univarite (no adjustment) and multivariate (adjusted for age and gender) linear regression models were applied to compare folate levels between the TD children and the ASD children. According to age, we divided the ASD children into four groups: ≤3 years, 3–4 years, 4–5 years, and >5 years. Then Spearman correlation analysis was used to explore the association of folate levels with symptoms and neurodevelopmental levels of ASD children at different age groups. Statistical software SPSS (SPSS 26.0, Inc., USA) was used to conduct the statistical analysis. In this study, a *p*-value <0.05 is considered to be statistically significant.

## Results

### Participant Demographic Characteristics Between TD and ASD Children

This study enrolled 1,300 autistic children, including 1,069 boys and 231 girls, with a median (IQR) age of 3.95 (3.12–4.90) years. The control group is comprised of 1,246 TD children, of which 819 were male and 427 were female, with a median (IQR) age of 4.42 (3.38–5.38) years. There were differences in the age distribution and the gender ratio between the two groups (*P* < 0.05). As shown in Table 1, the percentages of picky eating (59.54 vs. 34.29%), resistance to vegetables (28.46 vs. 18.49%), and resistance to meats (10.33 vs. 5.72%) in the children with ASD were significantly higher than those of the TD children (*P* < 0.05, respectively).

**Table 1:** Comparison of demographic characteristics between TD and ASD children.

**Variable**	**TD (*n* = 1,246)**	**ASD (*n* = 1,300)**	**Test**	***P***
Age (years), Median (IQR)	4.42 (3.38–5.38)	3.95 (3.12–4.90)	Z = −6.197	<0.001
Gender, *n* (%)				
Male	819 (65.73)	1,069 (82.23)	χ^2^= 90.382	<0.001
Female	427 (34.27)	231 (17.77)		
Ethnicity, *n* (%)				
Han	1,121(95.24)	1,170(93.53)	χ^2^= 3.358	0.067
Others	56 (4.76)	81 (6.47)		
Picky eating, *n* (%)	408 (34.29)	749 (59.54)	χ^2^= 156.464	<0.001
Resistance to vegetables, *n* (%)	220 (18.49)	358 (28.46)	χ^2^= 33.706	<0.001
Resistance to meats, *n* (%)	68 (5.72)	130 (10.33)	χ^2^= 17.454	<0.001

*Data was shown as Median (IQR) or number (percentage). Chi-square test and Mann-Whitney test were used in the analysis*.

*ASD, autism spectrum disorder; TD, typically developing; IQR, interquartile range*.

### Serum Folate Levels in TD and ASD Children

Previous research has reported that the variation in the level of serum folate is strongly relevant for ASD (14, 28). Therefore, we detected the levels of serum folate in the participants. The results of univariate (no adjustment) and multivariate (adjusting for age and gender) linear regression models are shown in Table 2. The unadjusted model showed that the serum folate levels of children with ASD were significantly lower than those of TD children (β = −0.540, 95% CI: −0.858~-0.222, *P* < 0.05). After adjusting for age and gender (adjusted model), the difference of folate between the two groups remained significant (β = −0.863, 95% CI: −1.166~-0.559, *P* < 0.05). Further gender-stratified analysis showed that children with ASD exhibited lower serum folate levels than TD children both in the male (β = −0.878, 95% CI: −1.233~-0.524, *P* < 0.05) and female groups (β = −0.813, 95% CI: −1.401~-0.226, *P* < 0.05) after adjusting for age (Table S1).

**Table 2:** Comparison of serum folate level between TD and ASD children in different models.

**Group**	**Folate_**(Median, IQR)**_**	**Unadjusted^a^**		**Adjusted^b^**	
		**β (95%CI)**	***P***	**β (95%CI)**	***P***
TD (*n* = 1,246)	12.32 (9.93, 15.13)	reference		reference	
ASD (*n* = 1,300)	11.53 (8.65, 15.09)	−0.540 (−0.858, −0.222)	0.001	−0.863 (−1.166, −0.559)	<0.001

*Data was presented as β (95%CI)*.

*Univariate linear regression was used for unadjusted model and multivariate linear regression was used for adjusted model*.

a *no adjustment*.

b *adjusted for age and gender*.

*TD, typically developing; ASD, autism spectrum disorder*.

*β (95%CI), regression coefficient (95% confidence interval); IQR, interquartile range*.

### Associations of Mealtime Behaviors, Age, and Gender With Serum Folate Levels in Autistic Children

**Figure 2:**
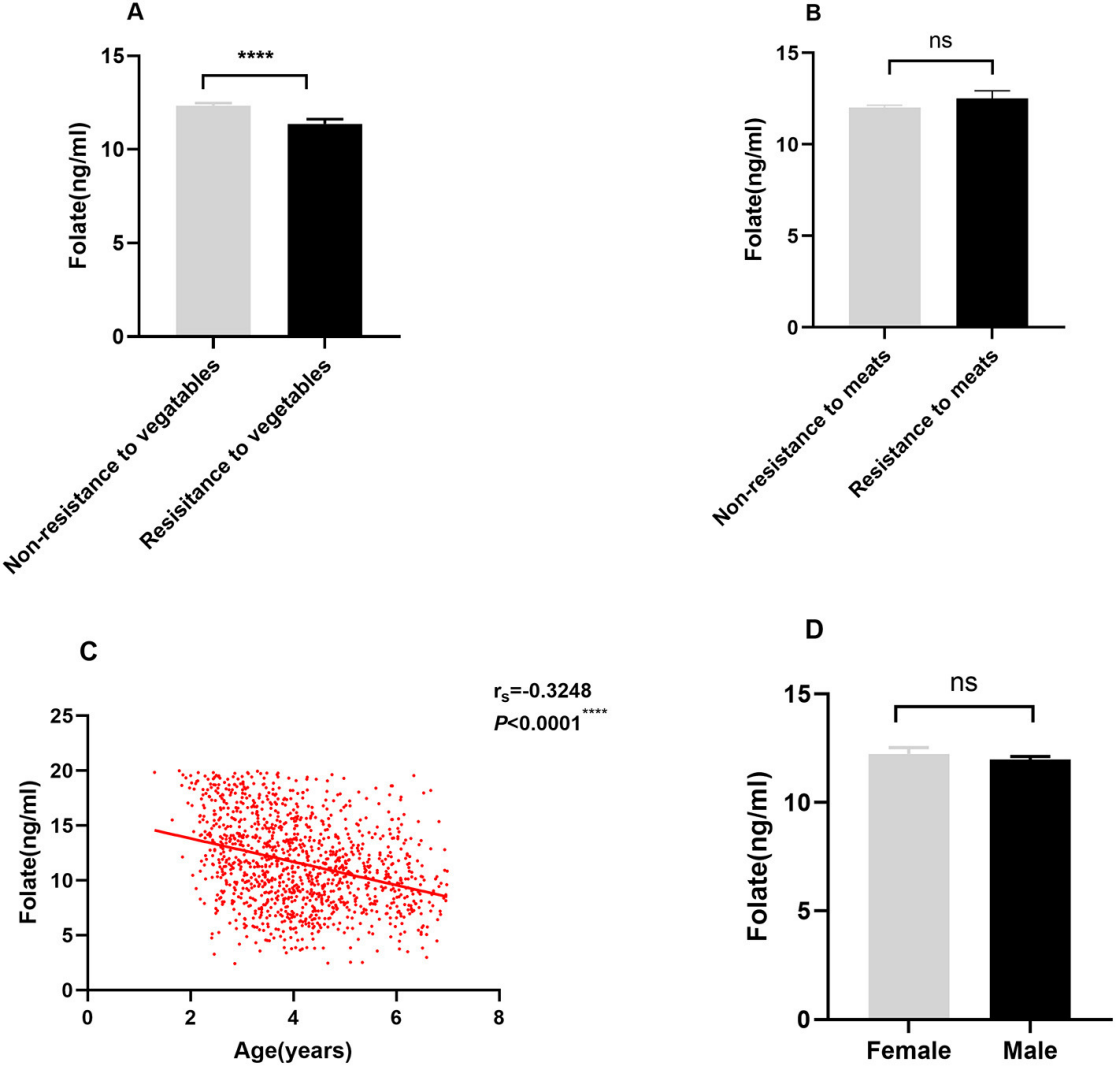
Associations of mealtime behaviors, age, and gender with serum folate level in autistic children. **(A,B)** Effect of mealtime behaviors on folate levels in ASD children; **(C)**: Correlation of folate levels with age; **(D)**: Comparison of folate levels between male and female children with ASD. Data was presented as Mean ± SEM, the Mann-Whitney test was used for the comparison. ****P < 0.0001, “ns” no significant difference.

Folate must be derived from the diet since the body cannot produce it *de novo*. Food rich in folate includes leafy vegetables, beans, nuts, and animal viscera. We further investigated the relationship between eating behaviors and serum folate levels. As shown in Figure 2A, serum folate levels were lower in autistic children with resistance to vegetables habit than those without this habit (*P* < 0.05). However, there were no significant differences in the serum folate levels of autistic children between the non-resistant to meats group and resistant to meats group (*P* > 0.05) (Figure 2B). We also examined the relationships between age and gender and serum folate levels in the ASD group. The result showed that age was negatively associated with serum folate levels (r_s_ = −0.3248, *P* < 0.05) (Figure 2C). However, there was no difference between gender and serum folate (*P* > 0.05) (Figure 2D).

### Associations Between Serum Folate Levels and ABC, SRS, CARS and Communication Warning Behavior of CNBS-R2016 Scores of Children With ASD at Different Age Groups

We divided the children with ASD into four groups by age: ≤3 years old, 3–4 years old, 4–5 years old, and >5 years old. Then we examined the associations between serum folate with ABC, SRS, CARS, and communication warning behavior of CNBS-R2016 scores at each age group. Our results showed that there were no significant correlations between serum folate levels and subscale of ABC, total ABC, subscale of SRS, total SRS, and total CARS scores at each age group (*P* > 0.05, respectively). However, serum folate level was negatively associated with communication warning behavior of CNBS-R2016 scores at the age of three and under (*P* < 0.05). In other age groups, no such association was observed (Table 3).

**Table 3:** Effect of serum folate level on ABC, SRS, CARS, and communication warning behavior score of CNBS-R2016 scores in the ASD children at different age groups.

**Item**	**≤3 y**		**3~4 y**		**4~5 y**		**>5 y**	
	**r_**s**_**	***P***	**r_**s**_**	***P***	**r_**s**_**	***P***	**r_**s**_**	***P***
**ABC(n1 = 253, n2 = 378, n3 = 299, n4 = 285)**								
Sensory	−0.078	0.234	0.085	0.118	−0.004	0.944	−0.085	0.172
Relating	0.01	0.881	0.105	0.054	0.111	0.076	−0.103	0.098
Body and object use	−0.067	0.309	−0.013	0.812	−0.031	0.617	−0.056	0.366
Language	−0.046	0.487	0.028	0.602	0.114	0.067	0.000	0.994
Social self-help	0.072	0.273	0.092	0.089	−0.001	0.998	−0.071	0.253
ABC total score	−0.002	0.980	0.082	0.113	0.056	0.338	−0.079	0.183
**SRS(n1 = 218, n2 = 313, n3 = 292, n4 = 285)**								
Social awareness	−0.079	0.247	−0.011	0.841	0.046	0.437	−0.053	0.376
Social cognition	−0.061	0.373	0.001	0.986	0.112	0.058	0.036	0.548
Social communication	−0.027	0.694	−0.026	0.646	0.105	0.074	0.000	0.997
Social motivation	−0.009	0.9	−0.018	0.751	0.066	0.266	−0.056	0.349
Autism behavior mannerisms	−0.06	0.376	−0.065	0.253	0.066	0.265	−0.031	0.608
SRS total score	−0.056	0.41	−0.037	0.516	0.101	0.087	−0.011	0.854
**CARS(n1 = 238, n2 = 337, n3 = 266, n4 = 264)**	−0.059	0.369	0.07	0.201	0.031	0.62	0.004	0.951
**Communication warning behavior (n1 = 177, n2 = 302, n3 = 238, n4 = 217)**	−0.193	0.011^*^	0.012	0.841	0.012	0.857	−0.012	0.858

*Data was presented as correlation coefficients of spearman. Spearman rank correlation was used in the analysis*.

* *P < 0.05*.

*y, years; n1, the sample of ASD children aged 3 years and under; n2, the sample of ASD children aged 3 to 4 years*.

*n3, the sample of ASD children aged 4 to 5 years; n4, the sample of ASD children aged 5 years after*.

### Associations Between Serum Folate Levels and Neurodevelopmental Levels of Children With ASD at Different Age Groups

**Figure 3:**
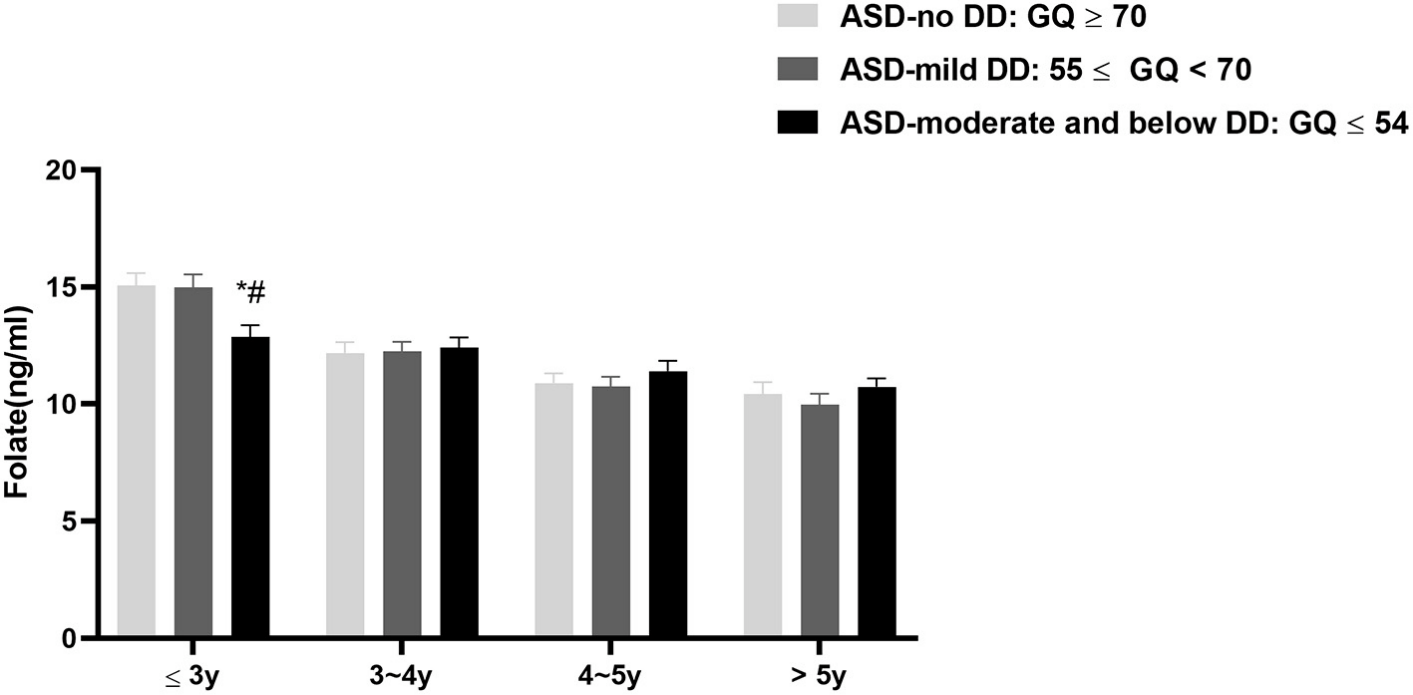
The serum folate levels among autistic children with different neurodevelopmental levels; Data was presented as mean ± SEM. The Kruskal-Wallis test was used for the comparison. *P < 0.05 vs. the ASD-no DD group, ^#^*P* < 0.05 vs. the ASD-mild DD group.

We also explored the associations between serum folate levels and neurodevelopmental levels of children with ASD at different age groups. Firstly, according to the general quotient (GQ), we divided the children with ASD into three subgroups: ASD-no DD: GQ ≥70; ASD-mild DD: 55 ≤ GQ <70; and ASD-moderate and below DD: GQ ≤ 54. Here the development quotient rank reference of mild DD as well as moderate and below DD was from the classification standard of Gesell Developmental Scale. We compared the serum folate levels among the three groups. The results showed that the serum folate level of ASD-moderate and below DD subgroup was lower than those of the ASD-no DD (adjusted *P* = 0.013) and the ASD-mild DD subgroups (adjusted *P* = 0.024) at the age of three and under. But there was no difference in serum folate levels between the ASD-no DD subgroup and ASD-mild DD subgroup (adjusted *P* > 0.05). In terms of other age stages, no significant differences were observed among the three groups (Figure 3).

Secondly, we analyzed the correlations between serum folate levels and development quotients at different ages. As is shown in Table 4, serum folate levels were positively associated with general quotient (r_s_ = 0.177, *P* = 0.018), gross motor quotient (r_s_ = 0.204, *P* = 0.006), fine motor quotient (r_s_ = 0.159, *P* = 0.035), and language quotient (r_s_ = 0.2, *P* = 0.008) at the age of three and under. However, at other age groups, no significant correlations were observed (*P* > 0.05).

**Table 4:** Effect of serum folate levels on CNBS-R2016 scores in the ASD children at different age groups.

**CNBS-R2016**	**≤3y(n=177)**		**3~4y(n=302)**		**4~5y(n=238)**		**>5y(n=217)**	
	**r_**s**_**	***P***	**r_**s**_**	***P***	**r_**s**_**	***P***	**r_**s**_**	***P***
GQ	0.177	0.018^*^	−0.054	0.347	−0.034	0.602	−0.047	0.489
Gross motor	0.204	0.006^**^	−0.06	0.298	−0.054	0.406	−0.053	0.436
Fine motor	0.159	0.035^*^	−0.057	0.326	−0.06	0.358	−0.059	0.387
Adaptive behavior	0.065	0.392	−0.068	0.237	−0.004	0.948	−0.015	0.829
Language	0.2	0.008^**^	−0.018	0.755	0.028	0.672	−0.025	0.709
Personal- social	0.119	0.114	−0.046	0.427	−0.091	0.162	−0.003	0.961

*Data was presented as correlation coefficients of spearman. Spearman rank correlation was used in the analysis*.

* *P < 0.05;*

** *P < 0.01*.

*CNBS-R2016, Children Neuropsychological and Behavior Scale-Revision 2016; GQ, general quotient*.

### Comparison of CNBS-R2016 Scores at Different Serum Folate Levels in ASD Children Aged Three and Under

**Figure 4:**
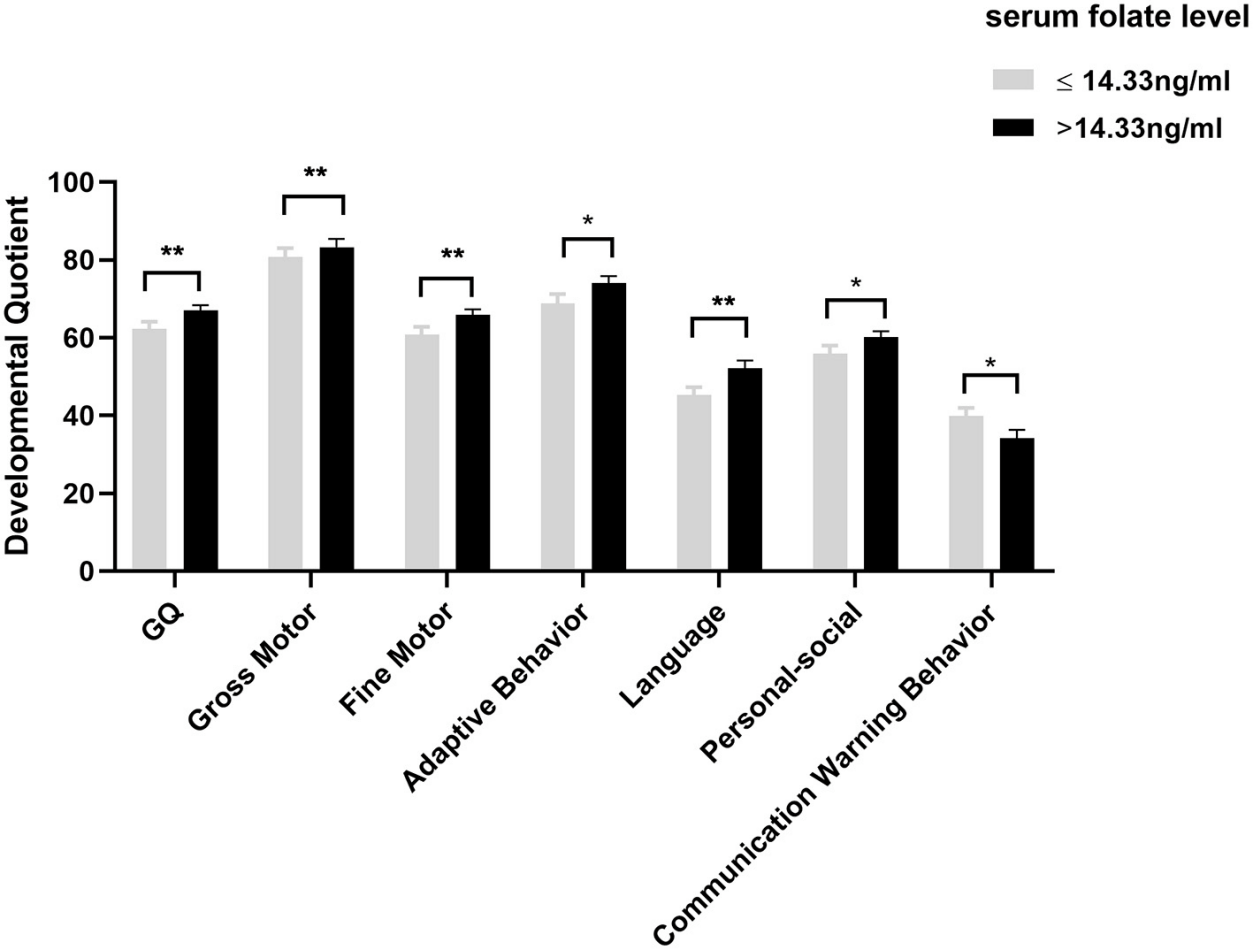
Comparison of CNBS-R2016 scores at different serum folate levels in ASD children aged three and under. Data was presented as mean ± SEM. The Mann-Whitney test and the two-tailed Student's *t*-test were used for the comparison. *P < 0.05, **P < 0.01.

So far, our reference criteria for folate deficiency comes from the reference values of WHO (< 4 ng/mL) (29) and domestic study in Guangdong (< 3.1 ng/mL), but these reference values come from adults. Consequently, there was no adequate value to judge whether children suffer from folate deficiency. As a result, according to the median of serum folate, we divided the autistic children aged three and under into two groups: folate ≤ 14.33 ng/mL group and folate >14.33 ng/mL group. We further analyzed the differences of CNBS-R2016 scores between the groups mentioned above. As shown in Figure 4, compared with patients with folate ≤ 14.33 ng/mL, patients with folate > 14.33 ng/mL had higher general quotient, gross motor quotient, fine motor quotient, adaptive behavior quotient, language quotient, and personal-social quotient (*P* < 0.05). In addition, the score of communication warning behavior in patients with folate > 14.33 ng/mL was lower than that in patients with folate ≤ 14.33 ng/mL (*P* < 0.05). These results suggest that at the age of three and under, relatively higher serum folate levels are more beneficial to cognition function development in children with ASD.

## Discussion

The etiology of ASD is not yet fully understood. While genetics is now a well-established risk factor, some data does support a contributing environmental factor well (6). Folate is an important environmental factor and can affect neurobiological development (30). Several studies have focused on the role of folate in ASD symptoms and neurodevelopment levels (14, 15, 31). However, these studies have not investigated the associations of folate and ASD symptoms and neurodevelopment levels stratified by age. In our study, we focused on the associations between serum folate level and the core symptoms and neurodevelopmental level of children with ASD at different age groups.

Folate is indispensable for normal neuronal function, and its serious deficiencies might directly affect brain function (32). Several studies have shown that the folate level of children with ASD differs from those of healthy children. Yahya M et al. found that compared with TD children, children with ASD have significantly lower folate levels in their diet and serum (33). However, there was another study that found children with ASD and healthy children differed significantly regarding vitamin B12 and homocysteine levels, but not folate levels (15). In our study, compared with TD children, children with ASD exhibited lower serum folate levels. Moreover, the serum folate levels in ASD children were still lower than those in TD children after gender stratification. Our result indicates that ASD children are more likely to have low folate levels. Some studies indicated that abnormal mealtime behaviors could cause nutritional vulnerability and consequently aggravate ASD symptoms (10, 34). Feeding problems, such as being a picky eater, lack of food variety, and resistance to new foods are more prevalent than in typically developing children. It is reported that children with ASD often prefer energy-dense foods such as meat, sugar-sweetened beverages, and snacks, and reject vegetables and fruits (34, 35). In our study, children with ASD exhibited more picky eating, especially resistance to vegetables, as compared with TD children. ASD children with a resistance to vegetables habit also exhibited lower serum folate levels, while such a difference was not observed in the autistic children with resistance to meats habit. It may be because folate cannot be produced by the body in *de novo*, and it must be derived from dietary sources and green leafy vegetables which contain the most folic acid. Additionally, our study found that age was negatively associated with serum folate levels and the serum folate levels were relatively high at younger ages. Similarly, the Helena Study showed that plasma folate concentrations decreased across age categories in European adolescents (36). Our results suggest that the serum folate status of autistic children may be associated with the intake of vegetables and the age of children.

Previous studies have explored the associations between folate and the symptoms of ASD. One study found that the CARS score of children with ASD was negatively associated with serum folate level (13). An open-label trial demonstrated that a 3-month folic acid intervention in autistic children participating in structured teaching significantly improved symptoms of autism (37). Since the clinical manifestations of children with ASD may be different at different ages (38), the associations between folate and ASD symptoms may vary by age. However, age-stratified analysis was not performed in these studies. In this study, we explored the relationship between serum folate and core symptoms and developmental levels of children with ASD by age stratification. After stratification, we found that, at each age group, serum folate levels were not associated with ABC, SRS, and CARS scores of children with ASD. However, the serum folate was negatively associated with the communication warning behavior score of CNBS-R2016 among children with ASD aged three and under. In terms of development quotients, those with ASD-moderate and below DD had lower serum folate levels than those in the ASD-noDD and ASD-mild DD at the age of three years and under. Further correlation analysis found that serum folate was positively associated with gross motor, fine motor, language, and general development quotients of children with ASD aged three years and under. These results suggest that the serum folate was closely related to the cognitive functioning of ASD children. Interestingly, Min Guo et al. also found that serum folate concentration was positively correlated with development quotients but was not correlated with ABC and SRS scores (14). Moreover, a study by Yektaş et al. reported that serum folate levels were not associated with CARS total and item scores (15). As a summary, the findings on the association of folate with ASD symptoms were inconsistent, possibly due to factors such as different ethnicities, different ability to assess symptoms by scales, and different age range of participants.

Our study also found that the association of serum folate with neurodevelopment levels and the communication warning behavior scores among ASD children was in the 3 years and under group rather than the other age groups which partially supported the results of some previous studies. For example, Remaekers et al. performed folinic acid treatment on children with low-functioning autism and with one or more of the major features of the infantile-onset cerebral folate deficiency (CFD) syndrome, and found that patients diagnosed early (2 and 3.2 years, respectively) had greater improvement in symptoms and the effect of folinic treatment on intellectual disability and ASD core symptoms decreased with advancing age (23). The American Academy of Pediatrics proposed that the most active period of neurodevelopment occurs in the first 1,000 days of life, during which nutrients such as iron, folate, and vitamin B12 are key factors in children's neurodevelopment and lifelong mental health (39). Folate provides methyl donors for phospholipids to form methylated phospholipids, which are required for the formation of myelination (40). The most significant period of myelination occurs from mid-gestation to age 2 years (41). Pujol et al. found that after 18 months, once the language brain attains the rapid myelination phase, children's language ability accelerates in vocabulary (42). Additionally, folate is essential for *de novo* purine and pyrimidine nucleotides synthesis to play a role in cell division and proliferation such as neural stem cells (43). The neuronal proliferation and differentiation are related to the formation of synapses, which peak in the 2nd and 3rd year after birth (44). Evidence proved that physiological processes such as neuron proliferation, myelination, and synapse formation are very important to the neural development of the brain (40). Therefore, during the rapid brain development phase, folate may affect neurodevelopment more easily, but the specific mechanism remains to be studied further.

Additionally, it should be noted that although the associations of folate with growth and development have been studied for decades, it is difficult to determine the true folate concentration in the low folate status among children because there is no folate-specific reference value for children. Therefore, we divided the patients aged 3 years and under into two groups according to the median level of serum folate, and compared the difference of development quotient at different serum folate levels in autistic children. The results showed that among autistic children aged 3 years and under, compared with the folate < 14.33 ng/mL group, the score of communication warning behavior was lower and the neurodevelopmental quotient in the folate ≥ 14.33 ng/mL group was higher. Our study firstly suggests that relatively higher folate levels may be more beneficial to neurodevelopment in autistic children. However, which concentration is most beneficial to the neurodevelopment of autistic children still needs to be studied further.

However, there exists some limitations in the present study. Firstly, the genders and ages of the two groups were not matched. Secondly, the transport of peripheral folate to the brain is related to folate receptors which can be blocked by folate receptor auto-antibodies (FRAA) (45), reduced folate carrier (43), mitochondria (46) and so on. If transport dysfunctions, such as the through the presence of FRAA, the folate in the brain will decrease. Therefore, peripheral blood folate level cannot fully reflect the central folate status. However, in our study, no indicators associated with central folate status except for blood folate level were detected due to the limitations of research centers. Thus, our study may not fully and accurately show the relationships between folate and ASD symptoms. Finally, our study was a cross-sectional design, so the conclusion may not be very affirmative. Prospective and randomized control trials are required to further observe the results after folic acid treatment.

## Conclusion

In the present study, we found that children with ASD had lower serum folate levels than typically developing children. Furthermore, serum folate was primarily associated with neurodevelopmental levels of children with ASD aged three and under. Relatively higher serum folate levels may be more beneficial to neurodevelopment in children with ASD. Therefore, it is necessary for children with ASD to actively evaluating folate status and ensure adequate folate level, especially in early life to better promote the intervention of children with ASD.

## Data Availability Statement

The raw data supporting the conclusions of this article will be made available by the authors, without undue reservation.

## Ethics Statement

The studies involving human participants were reviewed and approved by the ethics committee of the Children's Hospital of Chongqing Medical University, Approval Number: (2018) IRB (STUDY) NO. 121, and registered in the Chinese Clinical Trial Registry (Registration number: ChiCTR2000031194). Written informed consent to participate in this study was provided by the participants' legal guardian/next of kin.

## Author Contributions

QL, TY, L-JW, F-YJ, YH, LL, JZ, X-YK, M-JY, QH, J-JC, S-FF, Y-CW, QW, and C-HJ conducted data collection, process, and analysis. LC, YD, and T-YL performed the supervision of subjects' recruitment and collection at the clinic. JC and Z-FD performed the data interpretation and human samples supervision in the laboratory. TY, JC, and T-YL conceived, designed the study, and wrote the research protocol. LC, YD, JC, and T-YL performed the data interpretation and performed general supervision. QL, JC, and T-YL performed the data analysis, and drafted and revised the manuscript. All authors contributed to the article and approved the submitted version.

## Conflict of Interest

The authors declare that the research was conducted in the absence of any commercial or financial relationships that could be construed as a potential conflict of interest.

## References

[1] Lord C Elsabbagh M Baird G Veenstra-Vanderweele J. Autism spectrum disorder. Lancet. (2018) 392:508–20. 10.1016/S0140-6736(18)31129-2 30078460 PMC7398158

[2] Maenner M Shaw K Baio J Washington A Patrick M DiRienzo M. Prevalence of autism spectrum disorder among children aged 8 years - autism and developmental disabilities monitoring network, 11 Sites, United States, 2016. MMWR Surveill Summ. (2020) 69:1–12. 10.15585/mmwr.ss6706a1 32214087 PMC7119644

[3] Zhou H Xu X Yan W Zou X Wu L Luo X. Prevalence of autism spectrum disorder in China: a nationwide multi-center population-based study among children aged 6 to 12 years. Neurosci Bull. (2020) 36:961–71. 10.1007/s12264-020-00530-6 32607739 PMC7475160

[4] Bai D Yip B Windham G Sourander A Francis R Yoffe R. Association of genetic and environmental factors with autism in a 5-country cohort. JAMA Psychiatry. (2019) 76:1035–43. 10.1001/jamapsychiatry.2019.1411 31314057 PMC6646998

[5] De Rubeis S He X Goldberg A Poultney C Samocha K Cicek A. Synaptic, transcriptional and chromatin genes disrupted in autism. Nature. (2014) 515:209–15. 10.1038/nature13772 25363760 PMC4402723

[6] Emberti Gialloreti L Mazzone L Benvenuto A Fasano A Alcon AG Kraneveld A. Risk and protective environmental factors associated with autism spectrum disorder: evidence-based principles and recommendations. J Clin Med. (2019) 8:217. 10.3390/jcm8020217 30744008 PMC6406684

[7] Karhu E Zukerman R Eshraghi RS Mittal J Deth RC Castejon AM. Nutritional interventions for autism spectrum disorder. Nutr Rev. (2019) 78:515–31. 10.1093/nutrit/nuz092 31876938

[8] Wang C Geng H Liu W Zhang G. Prenatal, perinatal, and postnatal factors associated with autism: a meta-analysis. Medicine. (2017) 96:e6696. 10.1097/MD.0000000000006696 28471964 PMC5419910

[9] Modabbernia A Velthorst E Reichenberg A. Environmental risk factors for autism: an evidence-based review of systematic reviews and meta-analyses. Mol Autism. (2017) 8:13. 10.1186/s13229-017-0121-4 28331572 PMC5356236

[10] Liu X Liu J Xiong X Yang T Hou N Liang X. Correlation between nutrition and symptoms: nutritional survey of children with autism spectrum disorder in Chongqing, China. Nutrients. (2016) 8:294. 10.3390/nu8050294 27187463 PMC4882707

[11] Balashova O Visina O Borodinsky L. Folate action in nervous system development and disease. Dev Neurobiol. (2018) 78:391–402. 10.1002/dneu.22579 29380544 PMC5867258

[12] Main PA Angley MT Thomas P O'Doherty CE Fenech M. Folate and methionine metabolism in autism: a systematic review. Am J Clin Nutr. (2010) 91:1598–620. 10.3945/ajcn.2009.29002 20410097

[13] Altun H Kurutaş EB Sahin N Güngör O Findikli E. The levels of vitamin D, vitamin D receptor, homocysteine and complex B vitamin in children with autism spectrum disorders. Clin Psychopharmacol Neurosci. (2018) 16:383–90. 10.9758/cpn.2018.16.1.383 30466210 PMC6245292

[14] Guo M Li L Zhang Q Chen L Dai Y Liu L. Vitamin and mineral status of children with autism spectrum disorder in Hainan Province of China: associations with symptoms. Nutr Neurosci. (2020) 23:803–10. 10.1080/1028415X.2018.1558762 30570388

[15] Yektas C Alpay M Tufan AE. Comparison of serum B12, folate and homocysteine concentrations in children with autism spectrum disorder or attention deficit hyperactivity disorder and healthy controls. Neuropsychiatr Dis Treat. (2019) 15:2213–9. 10.2147/NDT.S212361 31496704 PMC6689552

[16] Tan M Yang T Zhu J Li Q Lai X Li Y. Maternal folic acid and micronutrient supplementation is associated with vitamin levels and symptoms in children with autism spectrum disorders. Reprod Toxicol. (2020) 91:109–15. 10.1016/j.reprotox.2019.11.009 31759952

[17] Nowakowski R. Stable neuron numbers from cradle to grave. Proc Natl Acad Sci U S Am. (2006) 103:12219–20. 10.1073/pnas.0605605103 16894140 PMC1567859

[18] Rakic P. No more cortical neurons for you. Science. (2006) 313:928–9. 10.1126/science.1131713 16917050

[19] Gilmore J Lin W Prastawa M Looney C Vetsa Y Knickmeyer R. Regional gray matter growth, sexual dimorphism, and cerebral asymmetry in the neonatal brain. J Neurosci. (2007) 27:1255–60. 10.1523/JNEUROSCI.3339-06.2007 17287499 PMC2886661

[20] Georgieff M Brunette K Tran P. Early life nutrition and neural plasticity. Dev Psychopathol. (2015) 27:411–23. 10.1017/S0954579415000061 25997762 PMC4443711

[21] McGovern C Sigman M. Continuity and change from early childhood to adolescence in autism. J Child Psychol Psychiatry. (2005) 46:401–8. 10.1111/j.1469-7610.2004.00361.x 15819649

[22] Koegel LK Koegel RL Ashbaugh K Bradshaw J. The importance of early identification and intervention for children with or at risk for autism spectrum disorders. Int J Speech Lang Pathol. (2014) 16:50–6. 10.3109/17549507.2013.861511 24328352

[23] Ramaekers VT Blau N Sequeira JM Nassogne MC Quadros EV. Folate receptor autoimmunity and cerebral folate deficiency in low-functioning autism with neurological deficits. Neuropediatrics. (2007) 38:276–81. 10.1055/s-2008-1065354 18461502

[24] Rellini E Tortolani D Trillo S Carbone S Montecchi F. Childhood Autism Rating Scale (CARS) and Autism Behavior Checklist (ABC) correspondence and conflicts with DSM-IV criteria in diagnosis of autism. J Autism Dev Disord. (2004) 34:703–8. 10.1007/s10803-004-5290-2 15679189

[25] Cen C Liang Y Chen Q Chen K Deng H Chen B. Investigating the validation of the Chinese Mandarin version of the Social Responsiveness Scale in a Mainland China child population. BMC Psychiatry. (2017) 17:51. 10.1186/s12888-016-1185-y 28166747 PMC5292795

[26] Schopler E Reichler R DeVellis R Daly K. Toward objective classification of childhood autism: Childhood Autism Rating Scale (CARS). J Autism Dev Disord. (1980) 10:91–103. 10.1007/BF02408436 6927682

[27] Li HH Feng JY Wang B Zhang Y Wang CX Jia FY. Comparison of the children neuropsychological and behavior scale and the griffiths mental development scales when assessing the development of children with autism. Psychol Res Behav Manag. (2019) 12:973–81. 10.2147/PRBM.S225904 31802957 PMC6801569

[28] Fuentes-Albero M Cauli O. Homocysteine levels in autism spectrum disorder: a clinical update. Endocr Metab Immune Disord Drug Targets. (2018) 18:289–96. 10.2174/1871530318666180213110815 29437021

[29] de Benoist B. Conclusions of a WHO Technical Consultation on folate and vitamin B12 deficiencies. Food Nutr Bull. (2008) 29:S238–44. 10.1177/15648265080292S129 18709899

[30] Lintas C. Linking genetics to epigenetics: the role of folate and folate-related pathways in neurodevelopmental disorders. Clin Genet. (2019) 95:241–52. 10.1111/cge.13421 30047142

[31] Schmidt R Hansen R Hartiala J Allayee H Schmidt L Tancredi D. Prenatal vitamins, one-carbon metabolism gene variants, and risk for autism. Epidemiology. (2011) 22:476–85. 10.1097/EDE.0b013e31821d0e30 21610500 PMC3116691

[32] Mitchell ES Conus N Kaput J. B vitamin polymorphisms and behavior: evidence of associations with neurodevelopment, depression, schizophrenia, bipolar disorder and cognitive decline. Neurosci Biobehav Rev. (2014) 47:307–20. 10.1016/j.neubiorev.2014.08.006 25173634

[33] Al-Farsi YM Waly MI Deth RC Al-Sharbati MM Al-Shafaee M Al-Farsi O. Low folate and vitamin B12 nourishment is common in Omani children with newly diagnosed autism. Nutrition. (2013) 29:537–41. 10.1016/j.nut.2012.09.014 23287069

[34] Evans EW Must A Anderson SE Curtin C Scampini R Maslin M. Dietary patterns and body mass index in children with autism and typically developing children. Res Autism Spectr Disord. (2012) 6:399–405. 10.1016/j.rasd.2011.06.014 22936951 PMC3427936

[35] Zhu J Guo M Yang T Lai X Tang T Chen J. Nutritional status and symptoms in preschool children with autism spectrum disorder: a two-center comparative study in Chongqing and Hainan Province, China. Front Pediatr. (2020) 8:469. 10.3389/fped.2020.00469 33014918 PMC7494825

[36] Gonzalez-Gross M Benser J Breidenassel C Albers U Huybrechts I Valtuena J. Gender and age influence blood folate, vitamin B12, vitamin B6, and homocysteine levels in European adolescents: the Helena Study. Nutr Res. (2012) 32:817–26. 10.1016/j.nutres.2012.09.016 23176792

[37] Sun C Zou M Zhao D Xia W Wu L. Efficacy of folic acid supplementation in autistic children participating in structured teaching: an open-label trial. Nutrients. (2016) 8:337. 10.3390/nu8060337 27338456 PMC4924178

[38] Hall AV Abramson R Ravan S Cuccaro ML Gilbert J Pericak-Vance M. Age and gender related symptom changes in children and adolescents with autism. In: 56th Meeting of American Academy of Child and Adolescent Psychiatry. Honolulu, HI.

[39] Schwarzenberg SJ Georgieff MK. Advocacy for improving nutrition in the first 1000 days to support childhood development and adult health. Pediatrics. (2018) 141:e20173716. 10.1542/peds.2017-3716 29358479

[40] Prado EL Dewey KG. Nutrition and brain development in early life. Nutr Rev. (2014) 72:267–84. 10.1111/nure.12102 24684384

[41] Naninck EFG Stijger PC Brouwer-Brolsma EM. The Importance of maternal folate status for brain development and function of offspring. Adv Nutr. (2019) 10:502–19. 10.1093/advances/nmy120 31093652 PMC6520042

[42] Pujol J Soriano-Mas C Ortiz H Sebastián-Gallés N Losilla J Deus J. Myelination of language-related areas in the developing brain. Neurology. (2006) 66:339–43. 10.1212/01.wnl.0000201049.66073.8d 16476931

[43] Desai A Sequeira JM Quadros EV. The metabolic basis for developmental disorders due to defective folate transport. Biochimie. (2016) 126:31–42. 10.1016/j.biochi.2016.02.012 26924398

[44] Nelson CA. A Neurobiological perspective on early human deprivation. Child Dev Perspect. (2010) 1:13–8. 10.1111/j.1750-8606.2007.00004.x

[45] Ramaekers V Segers K Sequeira J Koenig M Van Maldergem L Bours V. Genetic assessment and folate receptor autoantibodies in infantile-onset cerebral folate deficiency (CFD) syndrome. Mol Genet Metab. (2018) 124:87–93. 10.1016/j.ymgme.2018.03.001 29661558

[46] Rossignol D Frye R. A review of research trends in physiological abnormalities in autism spectrum disorders: immune dysregulation, inflammation, oxidative stress, mitochondrial dysfunction and environmental toxicant exposures. Mol Psychiatry. (2012) 17:389–401. 10.1038/mp.2011.165 22143005 PMC3317062

