# Supplementary material for: Serum Folate Status Is Primarily Associated With Neurodevelopment in Children With Autism Spectrum Disorders Aged Three and Under—A Multi-Center Study in China
Source: Front Nutr. 2021 May 13;8:661223. doi: 10.3389/fnut.2021.661223 (PMC8155683; doi:10.3389/fnut.2021.661223)
Supplement: Supplementary file 1 [file Table_1.docx]

| **Table S1.** Comparison of serum folate levels between TD and ASD children in different gender groups | | | | | |
| --- | --- | --- | --- | --- | --- |
| **Group** | **Unadjusted^a^** | |  | **Adjusted^b^** | |
|  | β(95%CI) | *P* |  | β(95%CI) | *P* |
| **Male** |  |  |  |  |  |
| TD(n=819) | reference |  |  | reference |  |
| ASD(n=1,069) | -0.528(-0.905, -0.151) | 0.006 |  | -0.878(-1.233, -0.524) | <0.001 |
| **Female** |  |  |  |  |  |
| TD(n=427) | reference |  |  | reference |  |
| ASD(n=231) | -0.449(-1.085, 0.186) | 0.166 |  | -0.813(-1.401, -0.226) | 0.007 |
| Data was presented as β(95%CI). Univariate linear regression was used for unadjusted model and multivariate linear regression was  used for adjusted model | | | | | |
| a: no adjustment | | | | | |
| b: adjusted for age | | | | | |
| TD: typically developing; ASD: autism spectrum disorder | | | | | |
| β(95%CI): regression coefficient (95% confidence interval) | | | | | |
